# Supplementary material for: Over-expression of TaDWF4 increases wheat productivity under low and sufficient nitrogen through enhanced carbon assimilation
Source: Commun Biol. 2022 Mar 3;5:193. doi: 10.1038/s42003-022-03139-9 (PMC8894359; doi:10.1038/s42003-022-03139-9)
Supplement: Supplementary file 2 — Supplementary Information [file 42003_2022_3139_MOESM2_ESM.pdf]

Suppl. Table 1: Percent Identity Matrix of *TaDWF4* genes in wheat compared to the rice *DWF4* (Os030227700)

	Percent identity							
Gene Locus	TraesCS3D01G526400	Os03g0227700-OsDWF4	TraesCS4B01G234100	TraesCS4A01G078000	TraesCS4D01G235200	TraesCS3A01G519000	TraesCS3B01G586500	TraesCS3D01G526300
TraesCS3D01G526400	100	80.52	83.96	84.43	84.2	89.86	87.97	90.07
Os03g0227700-OsDWF4	80.52	100	89.62	89.22	89.42	84.44	84.85	86.03
TraesCS4B01G234100	83.96	89.62	100	98.62	98.81	89.78	90.98	91.35
TraesCS4A01G078000	84.43	89.22	98.62	100	99.01	89.98	90.98	91.35
TraesCS4D01G235200	84.2	89.42	98.81	99.01	100	89.98	91.18	91.55
TraesCS3A01G519000	89.86	84.44	89.78	89.98	89.98	100	92.99	95.37
TraesCS3B01G586500	87.97	84.85	90.98	90.98	91.18	92.99	100	95.77
TraesCS3D01G526300	90.07	86.03	91.35	91.35	91.55	95.37	95.77	100

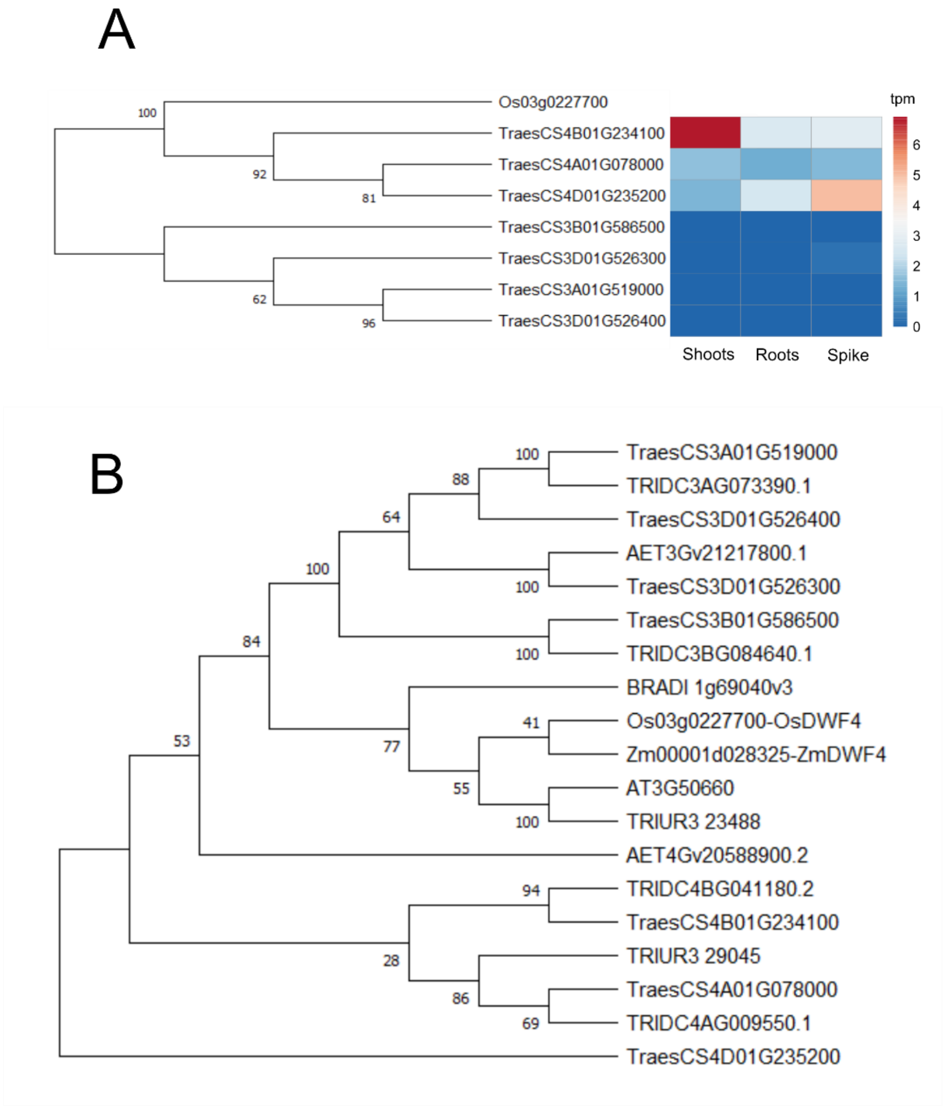

Suppl. Figure 1: Phylogenic tree *DWF4* genes in wheat and progenitor species. A) Phylogenic tree and expression levels in three tissues of the seven putative *DWF4* genes in wheat relative to the known *DWF4* gene from rice. Values on the tree represent the boot strap value from 500 iterations of aligning the sequences. Color code of the expression correspond to corresponding gene models based on the RefSeq v1 wheat genome. Expression values shown via heatmap of the seven putative *DWF4* genes in wheat cv. Chinese spring shown is tpm (transcripts per million). B) Phylogenitc tree of *DWF4* genes from wheat, rice, maize, *T.uratu, A.tauschii* and *T. dicoccoides*.

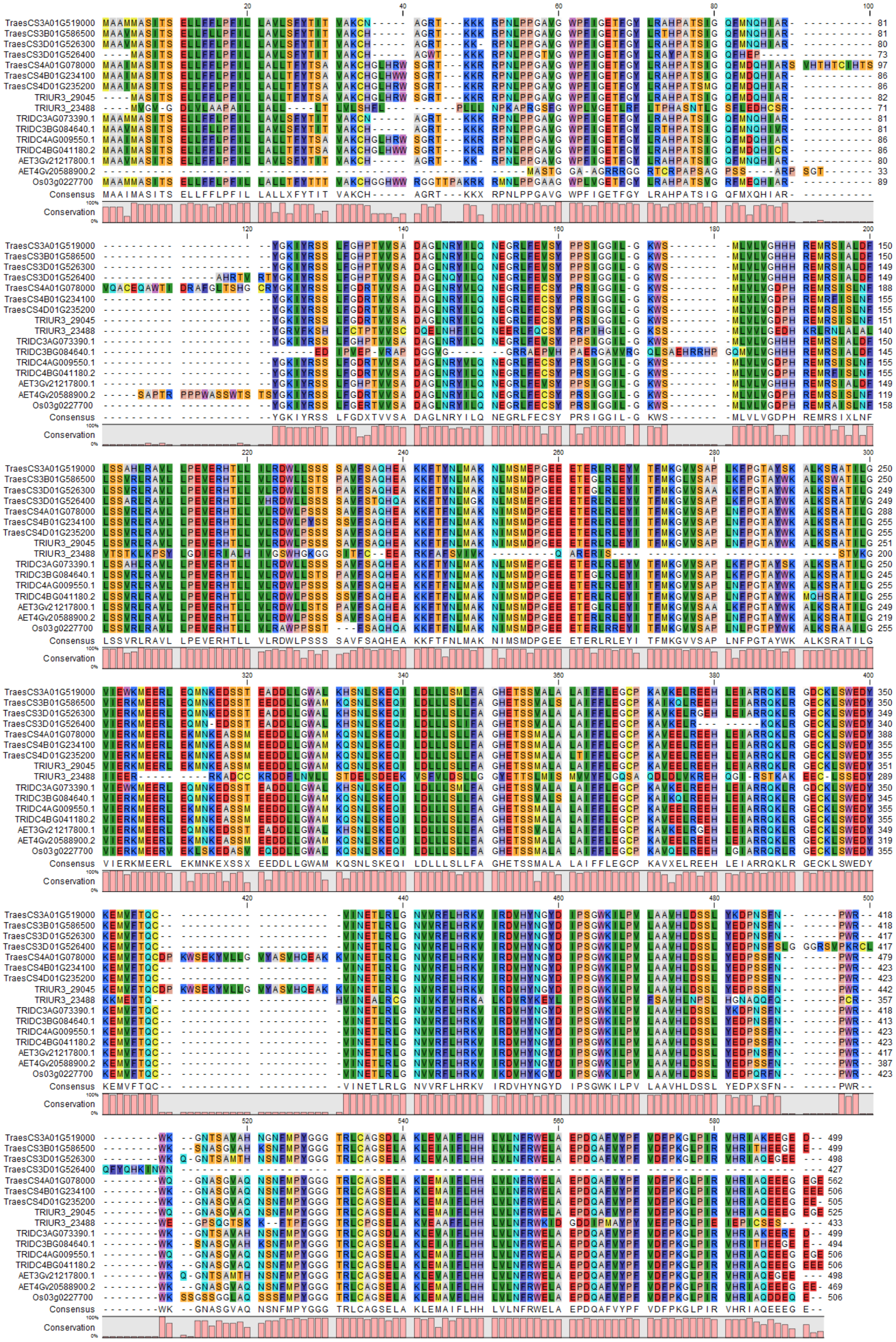

Suppl. Figure 2: Alignment of the amino acid sequences of the first isoform of each DWF4 homeologue from wheat and its progenitor species. Labels beginning with Traes are from bread wheat (refseq v1), TRIUR are from *T.uratu,* AET from *A.tauschii*, TRIDC from *T. dicoccoides,* Os Oryza sativa.

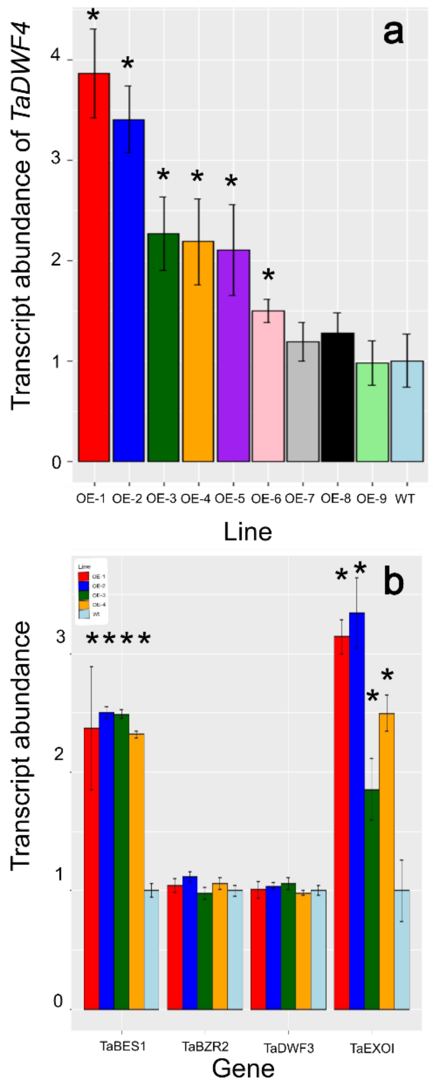

Suppl. Figure 3: Relative expression of *TaDWF4* and BR related genes as markers of BR levels in wheat over expression lines. A) *TaDWF4* expression in over expressing lines with a single copy T-DNA insertion. Expression shown is relative to *TaUbi*. B) Relative expression of *TaBES1, TaBZR2, TaDWF3 and TaEXOI* in the highest four *TaDWF4* over expressing lines with a single copy T-DNA insertion. Expression with SE is shown is relative to *TaUbi* from shoots of 14 day old plants. Significant differences are noted as * relative to WT expression (p val <0.05).

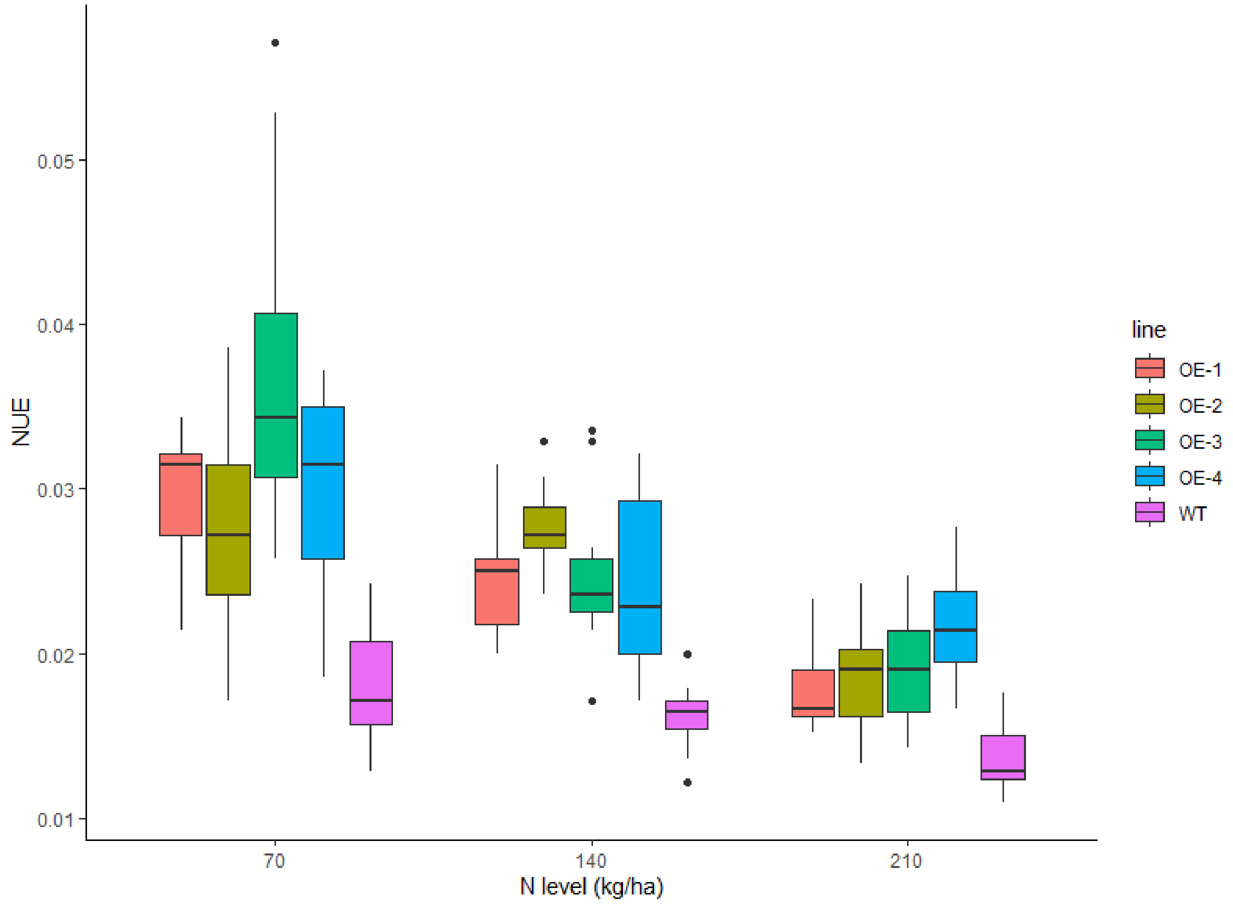

Suppl. Figure 4: NUE of *TaDWF4-B* overexpression lines grown under three different N concentrations

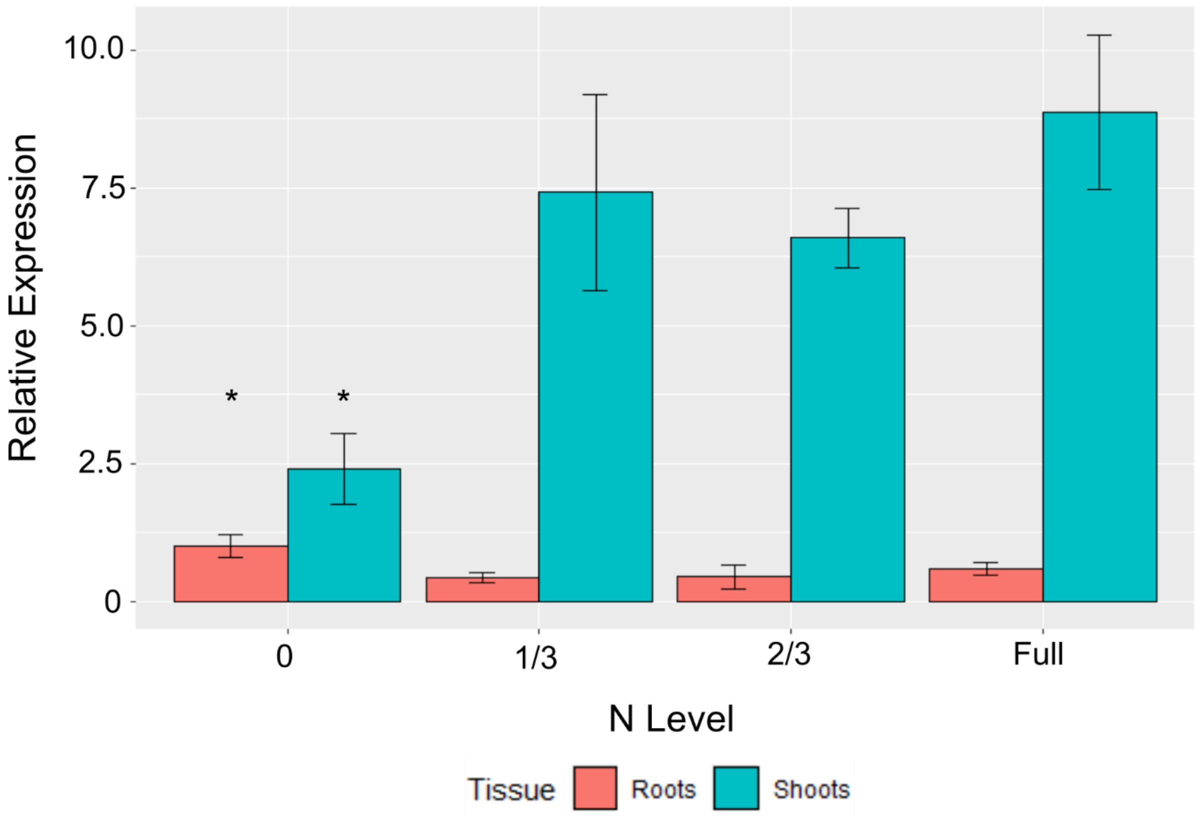

Suppl. Figure 5: Expression of *TaCYP90B* in Fielder wheat plants grown in hydroponics with various levels of N in solution roughly equivalent to 70, 140 and 210 kg/ha which equates to 1/3, 2/3 and Full N in hydroponics. Proportion of N is relative to the full strength solution. *TaDWF4* expression is shown relative to the *DWF4* expression grown with no N in the roots. Significant differences are noted as * (p val <0.05).

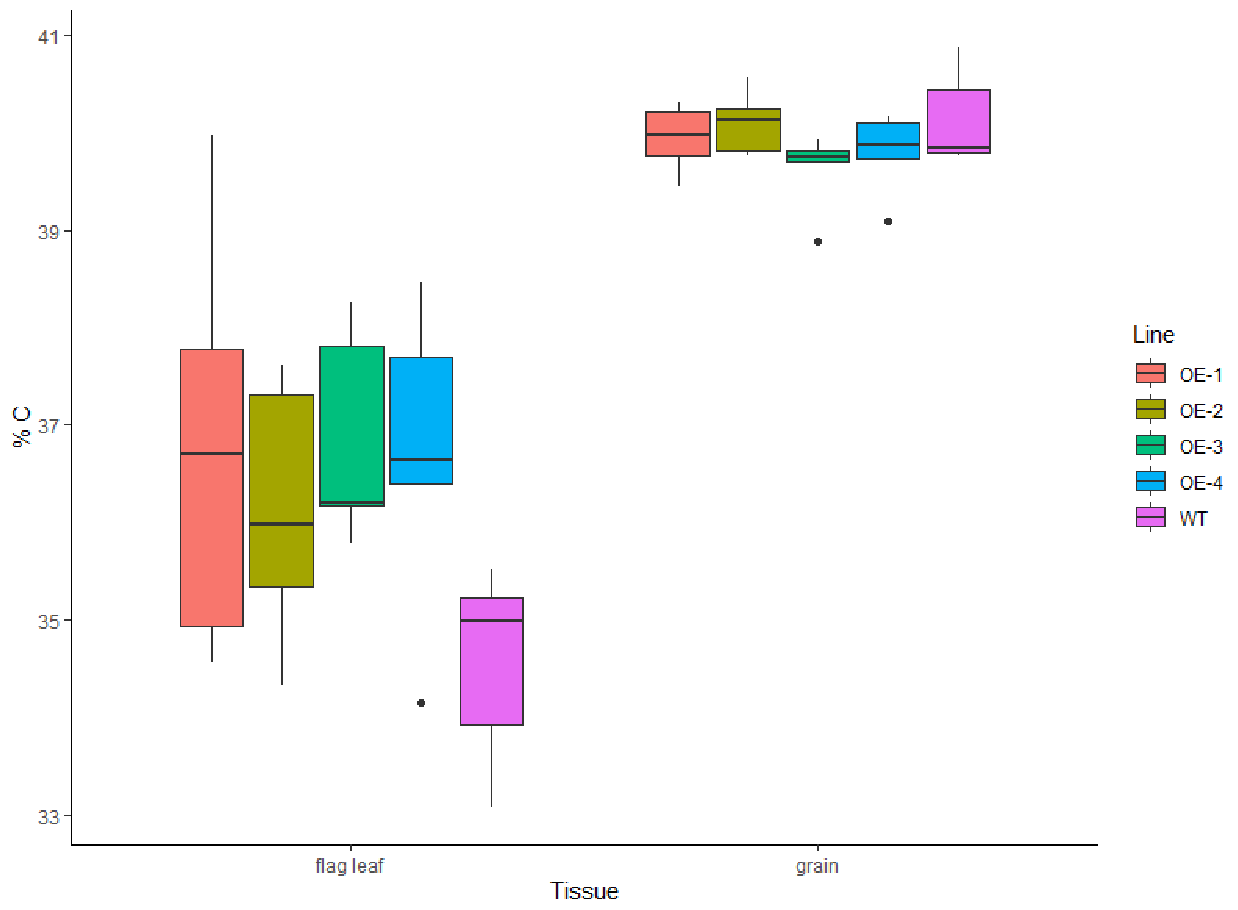

Suppl. Figure 6: Carbon content of flag leaves and grain.

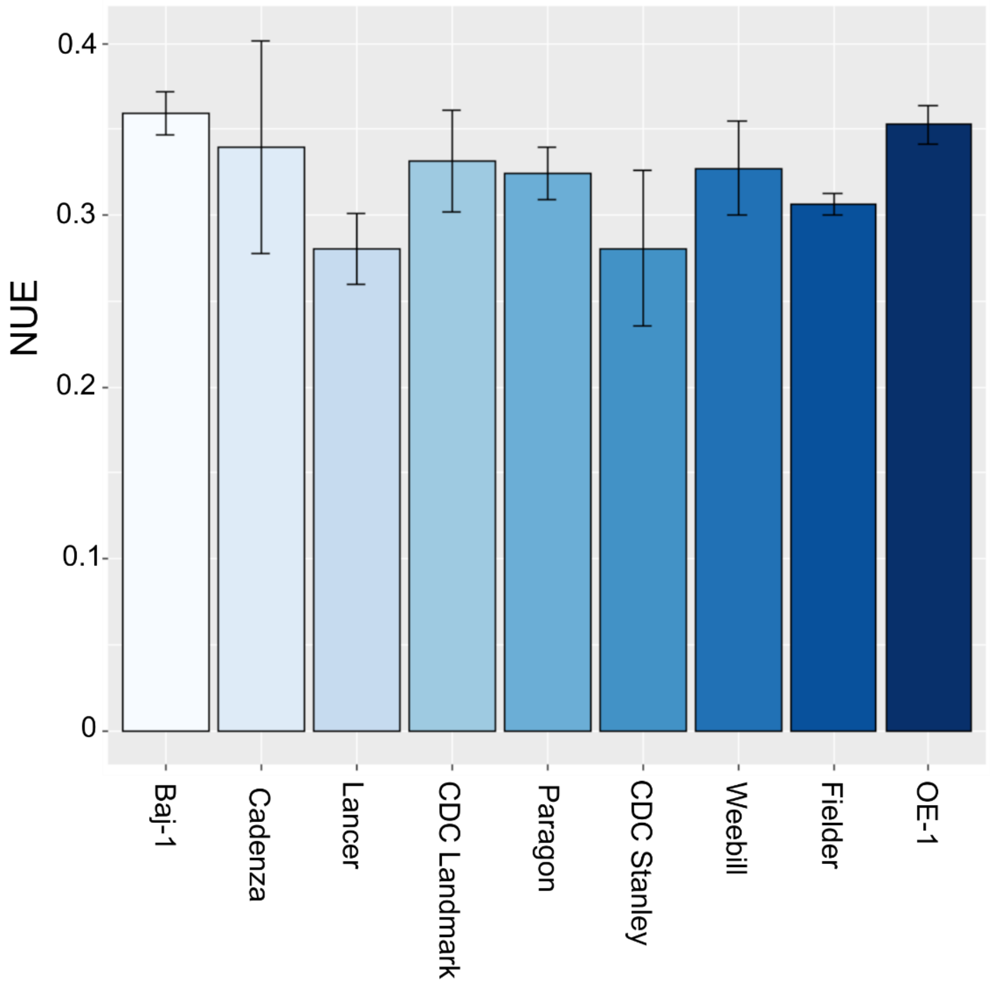

Suppl. Figure 7: NUE of spring wheat varieties grown on a low fertility soil supplemented with either 70 kg/ha (low) or 210 kg/ha (high). Data shown is the means of six plants for each treatment for the yield on low N divided by high N.
