# Supplementary material for: Over-expression of TaDWF4 increases wheat productivity under low and sufficient nitrogen through enhanced carbon assimilation
Source: Commun Biol. 2022 Mar 3;5:193. doi: 10.1038/s42003-022-03139-9 (PMC8894359; doi:10.1038/s42003-022-03139-9)
Supplement: Supplementary file 3 — Description of Additional Supplementary Files [file 42003_2022_3139_MOESM3_ESM.pdf]

Description of Additional Supplementary Files

File name: Supplementary Data 1

Description: Primers used in this study

File name: Supplementary Data 2

Description: TukeyHSD statistics for data in this study
